# Khat Chewing and Mental Distress: A Community Based Study, in Jimma City, Southwestern Ethiopia

**DOI:** 10.4314/ejhs.v21i1.69042

**Published:** 2011-03

**Authors:** Tekalign Damena, Andualem Mossie, Markos Tesfaye

**Affiliations:** 1 Department of Biomedical Sciences, College of Public Health and Medical Sciences, Jimma University, Jimma Ethiopia; 2 Department of Psychiatry, College of Public Health and Medical Sciences, Jimma University, P.O.Box 378, Jimma, Ethiopia

**Keywords:** Khat chewing, mental distress, mental illness, Self reporting questionnaire

## Abstract

**Background:**

Khat (Catha edulis) contains a psychoactive substance, cathinone, which produces central nervous system stimulation analogous to amphetamine. It is believed that khat chewing has a negative impact on the physical and mental health of individuals as well as the socioeconomic condition of the family and the society at large. There is lack of community based studies regarding the link between khat use and poor mental health. The objective of this study was to evaluate the association between khat use and mental distress and to determine the prevalence of mental distress and khat use in Jimma City.

**Methods:**

A cross-sectional community-based study was conducted in Jimma City from October 15 to November 15, 2009. The study used a structured questionnaire and Self Reporting Questionnaire-20 designed by WHO and which has been translated into Amharic and validated in Ethiopia. By multi stage sampling, 1200 individuals were included in the study. Data analysis was done using SPSS for window version 13.

**Results:**

The Khat use prevalence was found to be 37.8% during the study period. Majority of the khat users were males (73.5%), age group 18–24 (41.1%), Muslims (46.6%), Oromo Ethnic group (47.2%), single (51.4%), high school students (46.8%) and employed (80%). Using cut-off point 7 out of 20 on the Self Reporting Questionnaire-20, 25.8% of the study population was found to have mental distress. Males (26.6%), persons older than 55 years (36.4%), Orthodox Christians (28.4%), Kefficho Ethnic groups (36.4%), widowed (44.8%), illiterates (43.8%) and farmers (40.0%) had higher rates of mental distress. We found that mental distress and khat use have significant association (34.7% Vs 20.5%, P<0.001). There was also significant association between mental distress and frequency of khat use (41% Vs 31.1%, P<0.001)

**Conclusion:**

The high rate of khat use among the young persons calls for public intervention to prevent more serious forms of substance use disorders. Our findings suggest that persons who use khat suffer from higher rates of mental distress. However, causal association could not be established due to cross-sectional study design.

## Introduction

Khat *(Catha edulis)* contains a psychoactive substance, cathinone, which produces central nervous system stimulation analogous to amphetamine. It is widely used and highly praised in East Africa, including Ethiopia, and the Arabian Peninsula for its euphoric effect. The use is deeply anchored to regional customs and traditions (1).

College and University students also use it to stay alert, concentrate and have better flow of ideas. Perhaps, the central nervous system stimulant effect of cathinone is enhanced when it is combined with caffeine and cigarette smoking (2).

Prevalence of khat use among the staff of Jimma University and Jimma City community were found to be 30.75 % and 30.6 %, respectively (3,4). The prevalence of khat use reported for Adami Tulu was 31.7% and for Butajira was 50 % (5, 6). Similar studies among Mogadishu inhabitants in Somalia (7), and among students of Gondar College of Medical Sciences (8), had the prevalence of 18.3% and 22.3%, respectively.

There are few studies done on the relationship between khat use and mental distress. Some authors describe depression being associated with chewing khat, but almost all of these reports documented that depressive symptoms crop up on cessation of use. This has, on occasions, been associated with self-harm and suicide. Such behaviour has also been reported following amphetamine use and cessation (9). On the other hand, a study done on link between khat use and mental distress among homicide offenders did not show significant association (10).

Studies suggest that cathinone, which is an active ingredient of khat, increases heart rate, arterial blood pressure and respiratory rate transiently. It also improves cerebral blood flow, mental alertness and makes individuals energetic (11). In relation to this effect, regular khat chewing is thought to be a predisposing factor for gastritis and peptic ulcer disease, mental illness, cardiac arrhythmia, tooth decay and constipation. The mechanism of action of the active ingredient of khat, namely cathinone, is believed to be mediated via its sympathetic like action in the body (12).

Although the prevalence of khat use and its physiological and psychosocial effects are studied in some parts of Ethiopia, still there is limited knowledge on khat use and its mental distress level on the community. Because of this reason, it is imperative to do this research. Thus, this research would determine the prevalence of khat use and mental distress level and the association between the two.

## Materials and Methods

This cross-sectional community-based study was carried out from October 15, 2009 to November 15, 2009 in Jimma City, Oromia region, Southwest of Ethiopia 335km far from Addis Ababa. The city population is estimated to be 159,009 (13). Persons who were 18 years and above were invited to take part in the study.

Out of a total of thirteen kebeles of Jimma City, six were selected randomly using lottery method. Two hundred eighteen households were selected using systematic random sampling from each kebele. The households were selected from the list of house numbers; every third household was included in the survey. Once the household was identified, one individual selected randomly using lottery method from persons aged 18 or above living in that house. Using multi stage cluster sampling formula, prevalence rate of khat use (p) was taken to be 50% from previous study (6), with the confidence level of 95%, margin of error 0.03 and response rate of 82% a sample size of 1308 was required.

World Health Organization substance abuse questionnaire was adapted and modified to make it relevant to the objectives of the study (14). The magnitude of khat chewing and other parameters were addressed through this questionnaire. In addition, World Health Organization Self Reporting Questionnaire (SRQ) format which has been translated into Amharic and validated in Ethiopia was used to measure mental distress (15).

To identify potential problems and to make important modifications, the questionnaire was pre-tested on 20 randomly selected subjects who were not included in the study samples. Six trained graduate assistants were involved in filling the questionnaires during data collection. One day training was given for interviewers involved in the data collection. The reason for interviews as opposed to self administration was that some of the participants were not able to read and write.

The questionnaire was checked for its completeness and consistency during data cleaning and incomplete questionnaires were excluded. Then data analysis was made using SPSS for windows version 13.0. A participant was considered as having mental distress if he or she scored 7 or greater on SRQ-20.

Ethical clearance was obtained from Jimma university ethical review board (ERB) prior to data collection. The purpose of the study was explained and consent was obtained from the study subjects. In addition to this, confidentiality and anonymity were maintained by the investigator and research assistants throughout the study.

## Results

Data on 1200 out of 1308 subjects was available for analysis making an overall response rate of 91.7%. The reasons for the 108 (8.3 %) individuals who were not included in the study were: sixty did not consent for interview, 8 were unable to communicate and 40 had incomplete data. The majority (74 %) of the respondents were in the age group of 18–34 years with mean of 29.31 years. Over half (55.8 %) of the respondents were males, 48.5 % Orthodox Christians, 38.2 % Oromo, 28.3% employed. 49.1 % single and 89.2 % attended formal education.

Four hundred fifty three of the twelve hundred participants were chewing Khat giving the current prevalence rate 37.8%. Among khat users, males were 73.5%, Muslims 46.6%, and Oromo 47.2 %. One hundred forty nine (32.9%) of khat users were at high school level and 51.4% were single. Majority of chewers (41.1%) were found between the age group 18–24 years. One hundred twenty two (26.9 %) of the khat users were employees, 22.5% student, 21.0 % unemployed, 17.4% merchants and 1.8% were farmers (Table 1).

**Table 1:** Socio-demographic characteristics of Khat (Catha edulis) chewing and its prevalence in Jimma City, November 2009.

	*Population*		*Chewers*		*Non-chewers*			
*Variable*	*n*	*%*	n	%	n	%	*X*^2^	P-value
**Sex**								
Male	670	55.8	333	73.5	337	45.1	92.20	0.000
Female	530	44.2	120	26.5	410	54.9		
**Age**								
18–24	527	43.9	186	41.1	341	45.6		
25–34	361	30.1	159	35.1	202	27.0	21.98	0.000
35–44	184	15.3	79	17.4	105	14.1		
45–54	73	6.1	17	3.8	56	7.5		
55+	55	4.6	12	2.6	43	5.8		
**Religion**								
Orthodox	582	48.5	203	44.8	379	50.7		
Muslim	310	25.8	211	46.6	99	13.3	233.21	0.000
Protestant	259	21.6	21	4.6	238	31.9		
Catholic	19	1.6	1	0.2	18	2.4		
Others•	30	2.5	17	3.8	13	1.7		
**Ethnicity**								
Oromo	458	38.2	214	47.2	344	32.7		
Amhara	225	18.8	59	13.0	166	22.2	65.80	0.000
Gurage	119	9.9	67	14.8	52	7.0		
Kefficho	88	7.3	34	7.5	54	7.2		
Dawro	142	11.8	35	7.7	107	14.3		
Others§	168	14.0	44	9.7	124	16.6		
**Marital status**								
Married	507	42.3	183	40.4	324	43.4	8.92	0.030
Single	589	49.1	233	51.4	356	47.7		
Divorced	46	3.8	23	5.1	23	3.1		
Widowed	58	4.8	14	3.1	44	5.9		
**Educational level**	130	10.8	48	10.6	82	11.0		
Elementary	263	21.9	123	27.2	140	18.7	22.17	0.000
High school	399	33.3	149	32.9	250	33.5		
10+ 2	233	19.4	90	19.9	143	19.1		
10+ 3 & above	175	14.6	43	9.5	132	17.7		
**Occupation**								
Employee	339	28.3	122	26.9	217	29.0		
Unemployed	218	18.2	95	21.0	123	16.5	14.93	0.011
Merchant	194	16.2	79	17.4	115	15.4		
Farmer	10	0.8	8	1.8	2	0.3		
Student	308	25.7	102	22.5	206	27.6		
Othersψ	131	10.9	47	10.4	84	11.2		
**Total**	**120**		**45**		**74**			

• Waqefeta, atheists, Jehovah witness, Adventist, only Jesus…

§ Welita, Hadiya, Kembata, Tigre….

ψ Housewife, Retired….

**Figure 1:**
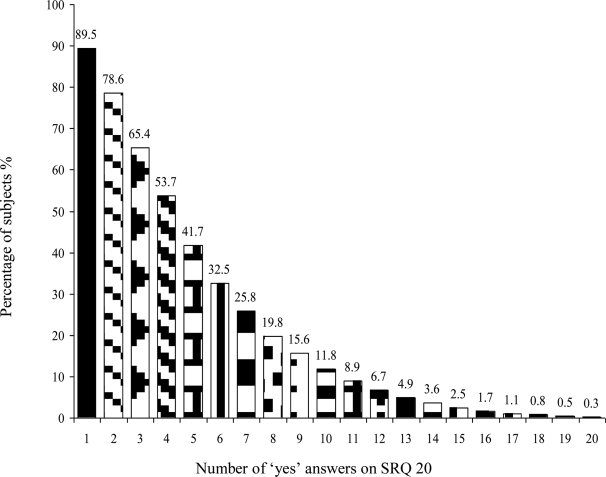
Mental distress symptom score on SRQ 20, Jimma City, November 2009

Over a quarter of the study participants (25.8%) were found to have mental distress (Fig. 1). Mental distress was found in 178 (26.6%) of male and 132(24.9%) of female participants. A relatively high prevalence (36.4%) of mental distress was found among Kefficho ethnic groups, Orthodox Christians (28.4%) and widowed (44.8%). Mental distress was 43.8%, 40% and 36.4% among illiterate, farmers and aged above fifty five years, respectively. Of all variables; age, religion, ethnicity, marital status, educational level and occupational status showed statistically significant association with mental distress (Table 2).

**Table 2:** Distribution and prevalence of *mental distress* according to socio-demographic characteristics, Jimma City, November 2009.

			***Mental distress***			
					
***Variables***		***Population***	**n**	**%**	***X*^2^**	***P-value***
**Sex**						
	Male	670	178	26.6	0.42	0.514
	Female	530	132	24.9		
**Age**						
	18–24	527	135	25.6		
	25–34	361	77	21.3		
	35–44	184	52	28.3	11.23	0.024
	45–54	73	26	35.6		
	55+	55	20	36.4		
**Religion**						
	Orthodox	582	165	28.4		
	Muslim	310	78	25.2		
	Protestant	259	54	20.8	9.51	0.049
	Catholic	19	2	10.5		
	Others•	30	11	36.7		
**Ethnicity**						
	Oromo	458	117	25.5		
	Amhara	225	48	21.3		
	Gurage	119	37	31.1	15.21	0.009
	Kefficho	88	32	36.4		
	Dawro	142	44	31.0		
	Others ψ	168	32	19.0		
**Marital status**						
	Married	507	115	22.7		
	Single	589	152	25.8	16.51	0.011
	Divorced	46	17	37.0		
	Widowed	58	26	44.8		
**Educational level**						
	Illiterate	130	57	43.8		
	Elementary	263	64	24.3		
	High school	399	107	26.8	29.56	0.000
	10+2	233	46	19.7		
	10+3 & above	175	36	20.6		
**Occupation**						
	Employee	339	73	21.5		
	Unemployed	218	75	34.4		
	Merchant	194	51	26.3		
	Farmer	10	4	40.0	13.18	0.022
	Student	308	75	24.4		
	Others§	131	32	24.4		
**Total**		**1200**	**310**	**25.8**		

• Waqefeta, atheists, Jehovah witness, Adventist, only Jesus…..

ψ Welita, Hadiya, Kembata, Tigre….

§ Housewife, Retired…

From a total of four hundred fifty three khat users, 157 (34.7%) had mental distress. Daily khat chewers and those who used khat for the last six months showed mental distress with a prevalence of 41.0% and 39.1%, respectively. Among the khat users with mental distress, 45.5% were smokers and 38.5% drank coffee during the chewing session and 43.9% took alcohol after chewing session. Khat use, frequency and duration of khat use had significant association with mental distress (P<0.001). Mental distress was significantly associated with those who were smoking and taking coffee during chewing and drank alcohol after chewing (P<0.001) (Table 3).

**Table 3:** Substance use and mental distress association, Jimma City, November 2009.

			***Mental distress***			
					
***Variables***		***Population***	**n**	**%**	***X*^2^**	***P-value***
Habit of Khat chewing						
	Yes	453	157	34.7	0.05	0. 000
	No	747	153	20.5		
Frequency of khat use						
	More than three days per week	166	68	41.0	38.49	0.000
	Less than four days per week	45	14	31.1		
Duration of khat use						
	≥ 2 years	136	49	36.0	162.21	0.000
	>2 years	317	108	34.1		
Smoking when khat chewing						
	Yes	110	50	45.5	20.69	0.000
	No	343	107	31.2		
Coffee when khat chewing						
	Yes	325	125	38.5	55.08	0.000
	No	128	32	25.0		
Alcohol after khat chewing						
	Yes	132	58	43.9	10.70	0.001
	No	321	99	30.8		

## Discussion

In this study, the current prevalence of khat use in Jimma City is lower than the one that was reported for Butajira (50%) (6). The Butajira study included largely rural community sample which might explain the difference from our study. But, the prevalence of khat use in this study is higher than the one reported from Jimma City (30.6%), and staff of Jimma University (30.75%) (4, 3). The former suggests that use of khat might be increasing in prevalence where as the latter is highly selected sample with high level of education as well as different cultural background.

In the current study, khat use was found to be more prevalent among males than females which is consistent with other studies done in Butajira and Jimma (6, 4). This may be due to the cultural and traditional restrictions on females to use khat and other substances. We also found significant association (P<0.001) between khat use habit and religion. Muslims were found to use khat more frequently than persons in other religions and this finding is similar to those reports from studies done in Agaro Secondary School students (16) Butajira (6) and Adamitulu (5). This might be due to the fact that khat growing and the practice of use have traditionally been confined to the Muslim population and the habit is socially accepted and could be easily passed from generation to generation (6).

This study reveals a strongly significant association (P<0.001) of khat use and Oromo ethnicity and the reason for this needs further investigation. Being single was found to be significantly associated with khat use (P<0.05). Furthermore, in this study, khat use and being student has been found to have statistically significant association (P=0.01) which is similar to findings from previous studies done in Jimma (11). The possible reason is that students use khat to stay alert and to get energy for their studies particularly during examination periods.

In Ethiopia, where there are great cultural and ethnic diversities, the need for using different cut-off points in different communities was apparent from previous studies. A cut-off point of 4/5 was initially used by Kortman (15) in an urban setting, which showed a prevalence of 12%. In a recent study conducted in Addis Ababa city in a larger sample using the SRQ a cut-off point of 6 out of 20 produce a prevalence estimate of 11.7% where as a study done on Butajira population using the same cut-off point yielded a high prevalence (45.3%). On the other hand studies from Jimma using a cut-off point of 6 and 7 showed prevalence rates of 22.7% and 35.9% respectively (10, 17).

In this study, the prevalence of 25.8% for mental distress is higher than those reported from previous studies in Ethiopia. Kortmann and Mulatu using the SRQ-20 reported a prevalence of 12% from Addis Ababa city and the town of Jimma (15, 18). However, the present finding (25.8%) is approximately consistent with the prevalence of mental distress in Ambo district which was 23.9% (19) and Jimma urban community which was 22.7 % (17), and lower than Jimma prison homicide offenders (35.9%) (10).

We found no significant association between mental distress and sex (P>0.05). Studies done in Africa and Ethiopia have reported an association between mental distress and female sex (20). A low social status, legal and/or economic discrimination, the affective nature of their response to stressors, hormonal changes and contraceptive pills have been incriminated as possible causes for the higher prevalence of depression and anxiety among women (6). This could probably due to the relatively small sample size of this study but needs further investigation. The positive association between older age and mental distress in this study is in agreement with studies done in Butajira (6), and Addis Ababa city (20). This could be explained by the tendency to have more symptoms owing to the probable accumulation of stressful life events and biological changes age increases.

Marital status was one of the factors which showed significant association with mental distress (P<0.05) where the married group experienced to a lesser extent mental distress than the widowed, divorced and singles in all age groups. Our finding contrasts with previous finding from Butajira (15) where singles suffered from a lesser degree of distress. Further investigations with better study designs are required to explain the findings.

Mental distress was significantly associated with khat use (P<0.001). Furthermore, there was significant association between mental distress and frequency and duration of khat use (P<0.001). This finding is in agreement with study done in Adami Tulu (5). It was higher among frequent and daily users and those who chewed for ≥ 2 years. Although causal relationship is difficult to establish using cross-sectional design, persons with mental distress might be using khat to alleviate their symptoms. In this study, mental distress was significantly (P<0.001) associated with khat users who drink alcohol. This result was similar to the findings from previous study in Ethiopia (6). A study done in Haromaya University students shows no association of mental distress and alcohol intake, however, the small sample size may have concealed the association in that study.

The apparent association between mental distress and use of coffee as well as smoking needs further investigation as there could be confounding between the variables. Nonetheless, this finding might also reflect the persons' attempt to treat their symptoms of anxiety and depression.

In conclusion large segment of the adult population of Jimma city have reported mental distress and may be suffering from mental disorders as well khat use appears to be increasing significantly in the city. Therefore, public health interventions aiming at reducing khat use need to be delivered to curb the increasing trend of khat use. In addition, policy makers should design strategies to control the production, usage and distribution of khat in Ethiopia. Enlightening mental health policy and legislation and training of professionals to control mental illness at the early stage are needed. Mental health services should be established for those who need them at all levels. Using this as a preliminary study, further investigation need to be conducted to explain the effect of chronic khat chewing on mental health.
